# Analysis of the regulatory mechanism of deoxynivalenol production using omics

**DOI:** 10.1186/s13568-018-0688-y

**Published:** 2018-10-03

**Authors:** Yumiko Iwahashi

**Affiliations:** National Agriculture and Food Research Organization, 2-1-2 Kannondai, Tsukuba, Ibaraki 305-8642 Japan

**Keywords:** *F. asiaticum*, Deoxynivalenol (DON), Omics, Glycolysis system, iTraq

## Abstract

*Fusarium* species are plant pathogens that produce various mycotoxins. Here, the regulatory mechanism of deoxynivalenol production in *Fusarium asiaticum* was analyzed using proteomic, metabolomic and transcriptomic methods. *F. asiaticum* was induced to produce deoxynivalenol by adding agmatine to the culture medium. Subsequently, metabolites of the glycolysis system were increased but mRNAs of the corresponding proteins were not up regulated. We speculated that this phenomenon was due to the up regulation of the 6-fructokinase and pyruvate kinase proteins, which are key enzymes of glycolysis. We discuss the relationship of metabolism with the regulation of deoxynivalenol production in *F. asiaticum.*

## Introduction

*Fusarium* species cause wheat and other small grain *fusarium* head blight (FHB) and corn ear rot in maize (Suga et al. 2008). There are at least 13 types of the *Fusarium* species at least physiologically different (Yli-Mattila et al. 2009), which produce mycotoxins, are a problem worldwide as plant pathogenic fungi, particularly because they decrease the yields of cereals. Among *Fusarium* species, *F. asiaticum* adapts to lower temperatures than another *Fusarium* species, preferring tropical climates, and can grow even at temperatures around 15 °C (Zhang et al. 2010). Therefore, *F. asiaticum* is widely distributed in Southeast Asia including Japan. In Japan, there are many areas where rain falls in the latter part of the wheat growing season and the final production tends to be damaged by mold contamination.

**Fig. 1:**
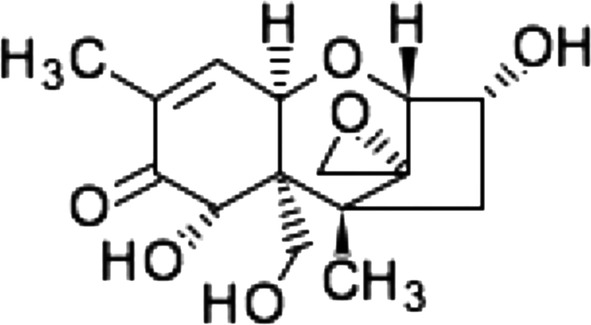
Chemical structure of deoxynivalenol (DON)

Various kinds of mycotoxins are produced by *Fusarium* species. Among them, deoxynivalenol (DON) is known to have acute toxicity, causing major disorders of the gastrointestinal tract such as vomiting and diarrhea (Pestka and Smolinski 2005; Chu 1998; Larsen et al. 2004). DON is sesquiterpenes with a trichothecene ring (Fig. 1). It is difficult that this chemical substance to completely inactivate in ordinary processing and cooking steps (Sugita-Konishi et al. 2006), so it is particularly important to prevent contamination at the production stage. Because of current food control policies in developed countries, DON pollution hardly ever causes acute toxicity. However, it is unknown whether long-term intake of low concentrations can be prevented and, especially in domestic animals and children, there is concern about the effects of DON including weight loss and reduction of immunity. In a study in Britain, DON was detected in the urine of > 95% of children and adolescents (Papageorgiou et al. 2018).

Cereals contaminated with fungi cannot be distributed, so when contaminated crops are accidentally mixed with harvests from other fields, all of the cereals, including the healthy ones, must be disposed of, resulting in economic losses.

The amount of DON produced varies depending on the environment and the state of the plant host. However, the regulatory mechanism of DON production is not well understood.

*Fusarium asiaticum* strain MAFF111889, which we used for this experiment, does not normally produce DON in modified Czapek liquid medium (pH 7.7) but does produce DON when agmatine is added to the medium. *F. asiaticum* induced to produce DON by adding agmatine shows increases in many metabolites and mRNAs (Suzuki et al. 2013).

A proteomice analysis method using isobaric tags for relative and absolute quantitation (iTraq) was published in 2004 (Ross et al. 2004) and can comprehensively compare the abundances of proteins among multiple samples. In the iTraq method, tags that can be distinguished from each other are made by replacing some atoms with stable isotopes without changing the molecular structure of the reporter group. The balance group also uses stable isotopes and is adjusted so that the molecular weight of the entire tag does not change. Thus, four or eight distinguishable tags are included in the iTraq reagent with the same structure, and the same mass as a whole.

Using iTraq, we found that three enzymes of the glycolysis system were up regulated when agmatine was added to induce DON production. In particular, 6-fructokinase and pyruvate kinase behaved very similarly to way they do in the metabolism of cancer cells. In recent years, metabolic fluctuations and posttranslational modifications have been clearly linked to human cancer cells (Hitosugi and Chen 2014). In particular, the regulation of metabolism by posttranslational modification mechanisms has attracted attention. Here, we discuss the changes in metabolites and posttranslational modifications in the regulation of DON production in *F. asiaticum.*

## Materials and methods

### Stocks, media, growth conditions and chemical substances

*Fusarium asiaticum* strain MAFF111889 was used in this study. This strain produces DON but does not produce nivalenol. The strain was maintained on potato dextrose agar purchased from Becton–Dickinson (Sparks, MD, USA). To determine the growth rate and DON yield, the strain was cultured in modified Czapek liquid medium (pH 7.7) containing 30.0 g/l sucrose, 2.0 g/l NaNO_3_, 15 mM MgSO_4_ and H_2_O (pH 7.7). Agmatine was purchased from Wako Pure Chemical (Osaka, Japan). Conidia or hyphae of *F. asiaticum* were inoculated into potato dextrose liquid medium and preincubated at 25 °C and 100 rpm for 3 days. The conidia or mycelia of the preincubated sample were collected by filtration and 100 mg were inoculated into medium with or without 2 mM agmatine and incubated at 25 °C for 5 days at 100 rpm.

### Determination of DON yield

The DON concentration in the culture filtrate was determined using the Veratox DON ELISA kit (Neogen Corporation, Lansing, MI, USA) according to the manufacturer’s instructions. Culture filtrates were initially diluted 1:10 in phosphate-buffered saline (pH 7.5) to provide a detection range of 0.5–6 ppm. Further sequential 1:10 dilutions were made to quantify toxin levels in samples that contained levels above the detection range.

### Proteins sample preparation

Culture filtrates (1 g) were frozen in liquid nitrogen and crushed to a fine powder. Then, 1 ml of 10 mM Tris–HCl (pH 7.5) was added and the sample was centrifuged at 2000*g* for 15 min. The centrifuged supernatant was treated with 1000 U of nuclease (Takara Bio Inc., Shiga, Japan) at 4 °C, and thereafter acetone precipitation was repeated four times at − 20 °C. The precipitate was recovered by centrifugation at 2000*g *for 15 min and suspended in a rehydration buffer (GE Healthcare UK Ltd., Buckinghamshire, England) to prepare a protein sample.

### Proteome analysis

The protein samples (50 μg each) were acetone-washed, reduced, alkylated and trypsin-digested according to the iTraq protocol (Sciex, Framingham, MA, USA). All labelled samples were combined to make a pooled sample. The peptides were desalted on a Strata-X 33-μm polymeric reversed phase column (Phenomenex, Torrance, CA, USA) and dissolved in a buffer containing 2% acetonitrile and 0.1% formic acid before separation by high pH on an Agilent 1100 high-performance liquid chromatography system (HPLC) using a Zorbax C18 column (2.1 × 150 mm, Agilent Technologies, Palo Alto, CA, USA). The peptides were eluted with a linear gradient of 20 mM ammonium formate, 2% acetonitrile to 20 mM ammonium formate, and 90% acetonitrile at 0.2 ml/min. The 95 fractions were concatenated into 12 fractions and dried. Each fraction was analyzed by electrospray ionization mass spectrometry using the Shimadzu Prominence nano HPLC system (Shimadzu Corporation, Kyoto, Japan) coupled to a 5600 Triple time-of-flight mass spectrometer (Sciex). The peptides were loaded onto a Zorbax 300SB-C18 3.5-μm column (Agilent Technologies) and separated with a linear gradient of water/acetonitrile/0.1% formic acid (v/v). Two types of samples, + agmatine and control, were used, and 2 × 4 (independent experimental samples) = 8 samples were analyzed simultaneously.

### Data analysis

Spectral data were analyzed using the ProteinPilot™ 5.0 Software (Sciex). The results were searched against the UniProt *F. graminearum* database (Version: August 2017; 16,432 sequences), a comprehensive, audited database designed specifically for mass spectrometry applications. For proteins that could not be identified with UniProt, the KEGG database was also used as a reference. The ProteinPilot™ Software calculates a percentage of confidence that reflects the probability that the hit is a false positive. At a 95% confidence level, there is a false positive identification rate of around 5%.

### Transcriptomic and metabolomic analysis

The expression level ratios of mRNAs and the content ratios of metabolites were measured as described in a previous paper (Suzuki et al. 2013).

## Results

### Proteomic analysis by liquid chromatography coupled with tandem mass spectrometry (LC–MS/MS) using iTraq reagent

LC–MS/MS with the iTraq reagent was used for comparative analysis of the intracellular proteins from *F. asiaticum* cultured with and without 2 mM agmatine. According to a *t* test, 506 peptides were identified with a p-value of 0.05 or less. Among them, 174 peptides were found in *F. asiaticum* cells less than 2 mM agmatine, which produced DON and contained over 1.5 times more protein than the control. Compared with the control, 135 peptides had decreased intracellular content less than 2 mM agmatine. Furthermore, 460 peptides were compared with DNA microarrays results. About 78% of protein and mRNA ratios were consistent (data not shown).

### Glycolysis system

Among the enzymes belonging to the glycolytic system, the peptide contents of three proteins, ATP-dependent 6-phosphofructokinase, 2,3-bisphosphoglycerate-independent phosphoglycerate mutase and pyruvate kinase, were increased by the addition of agmatine (4.4–6 times). ATP-dependent 6-phosphofructokinase and pyruvate kinase are considered to control the flow of glycolysis system. The mRNA content ratios were 0.6–1.48 by the addition of agmatine (Table 1).

**Table 1:** Proteomic and transcriptomic analysis of enzymes belonging to the glycolysis system

Accession #	FGSG	Protein peptide		mRNA*	Description
		*p*-value (agmatine ±)	Fold change (agmatine ±)	Fold change (agmatine ±)	
tr|I1RA62|I1RA62_GIBZE	FG00387.1	0.0457	0.8318	1.18	Phosphoglucomutase
tr|I1RP86|I1RP86_GIBZE	FG05843.1	0.0397	1.3677	1.03	Glucose-6-phosphate isomerase
tr|I1RYK0|I1RYK0_GIBZE	FG09456.1	0.01	4.6132	1.48	ATP-dependent 6-phosphofructokinase
tr|I1RGB2|I1RGB2_GIBZE	FG02770.1	0.0161	0.9376	0.74	Fructose-bisphosphate aldolase
tr|V6RLM7|V6RLM7_GIBZE	FG06702.1	0.0401	1.2359	1.10	Triose-phosphate isomerase
tr|V6R5I4|V6R5I4_GIBZE	FG03992.1	0.0456	1.0186	1.01	Phosphoglycerate kinase
tr|A0A098E4T8|A0A098E4T8_GIBZE	FG06055.1	0.0058	6.0813	0.61	2,3-bisphosphoglycerate-independent phosphoglycerate mutase
tr|I1RCM5|I1RCM5_GIBZE	FG01346.1	0.0003	1.0375	0.99	2-phosphoglycerate dehydratase
tr|I1RTL7|I1RTL7_GIBZE	FG07528.1	0.006	4.4055	0.86	Pyruvate kinase

Protein peptides were detected using LC–MS/MS as described in the “Materials and methods” section. The data marked with asterisk (*) are quoted from Suzuki et al. (2013)

### Proteomic, metabolomic and transcriptomic changes related to methionine

Methionine metabolites include methionine, S-adenosylmethionine, S-adenosylhomocysteine, homocysteine, cystathionine and cysteine. Of these, the S-adenosylmethionine, S-adenosylhomocysteine and cystathionine contents were increased 1.6- to 2.7-fold (Table 2). Homocysteine and cysteine could not be detected. In addition, *S*-adenosylmethionine synthetase, adenosylhomocysteinase and homocysteine methyltransferase peptides were up regulated when agmatine was added, but cystathionine beta-synthase was down regulated in both the transcriptome and proteome (Table 3).

**Table 2:** Changes in metabolites of the methionine cycle after addition of agmatine

Compound name	KEGG ID	Agmatine+/agmatine−	
		Ratio	*p*-value
Methionine	C00073,C00855,C01733	1.9	0.034
*S*-Adenosylmethionine	C00019	2.0	0.010
*S*-Adenosylhomocysteine	C00021	2.7	0.008
Homocysteine	C05330	ND	ND
Cystathionine	C00542,C02291	1.6	0.002
Cysteine	C00097,C00736,C00793	ND	ND

The content ratios of metabolites were measured as described in a previous paper (Suzuki et al. 2013)

**Table 3:** Proteomic and transcriptomic analysis of enzymes belonging to methionine cycle after addition of agmatine

Accession #	FGSG	Protein		mRNA	Description
		*p*-value (agmatine ±)	Fold change (agmatine ±)	Fold change (agmatine ±)	
tr|I1RA94|I1RA94_GIBZE	FG00421.1	0.0489	1.2023	1.82	*S*-Adenosylmethionine synthetase
tr|A0A0E0SID1|A0A0E0SID1_GIBZE	FG05615.1	0.0450	1.0471	2.46	Adenosylhomocysteinase
tr|I1S241|I1S241_GIBZE	FG10825.1	0.0446	0.9817	1.56	Homocysteine methyltransferase
tr|V6RDH3|V6RDH3_GIBZE	FG06544.1	0.0459	0.6194	0.80	Cystathionine beta-synthase

Protein peptides were detected using LC–MS/MS as described in the “Materials and methods” section. Accession # is the number in UniProt. Data marked with an asterisk (*) are quoted from Suzuki et al. (2013)

### TCA cycle, GABA shunt and glyoxylic acid cycle

Many of the proteins belonging to the TCA cycle were more down regulated than the corresponding mRNAs by addition of agmatine. In addition, pyruvate dehydrogenase, which produces acetyl-CoA from pyruvate in mitochondria, was down regulated by agmatine in both proteomic and transcriptomic analysis (Table 4). Conversely, the mRNA content of pyruvate dehydrogenase kinase, which phosphorylates pyruvate dehydrogenase and inhibits the influx of pyruvate into mitochondria, was up regulated. However, pyruvate dehydrogenase kinase could not be detected by proteomic analysis. Of the proteins belonging to the TCA cycle, only the isocitrate dehydrogenase protein was up regulated by agmatine addition; the expression ratio of the mRNA was also up regulated. ATP citrate lyase, which converts citrate from the mitochondrial matrix into cytoplasmic acetyl-CoA, was increased about 3.3-fold for the peptide and 2.8-fold for the mRNA. Furthermore, the abundance ratio of the isocitrate lyase protein of the glyoxylic acid cycle was increased by agmatine, which was similar to the mRNA.

**Table 4:** Proteomic and transcriptomic analysis of enzymes of TCA cycle and the GABA shunt

Accession #	FGSG	Protein peptide		mRNA*	Description
		*p*-value (agmatine ±)	Fold change (agmatine ±)	Fold change (agmatine ±)	
tr|I1RN88|I1RN88_GIBZE	FG05454.1	0.0421	0.8318	1.50	Pyruvate dehydrogenase
tr|I1RUQ3|I1RUQ3_GIBZE	FG07953.1	0.0499	0.8091	1.39	Aconitate hydratase
tr|I1RNY6|I1RNY6_GIBZE	FG05733.1	0.0171	3.1623	2.05	Isocitrate dehydrogenase
tr|I1RKB2|I1RKB2_GIBZE	FG04309.1	0.467	1.4322	1.44	2-oxoglutarate dehydrogenase
tr|I1REE2|I1REE2_GIBZE	FG02030.1	0.0191	0.8395	1.30	Succinate-coa ligase
tr|V6RRM0|V6RRM0_GIBZE	FG08712.1	0.0443	0.912	0.83	Fumarate hydratase
tr|I1RFI4|I1RFI4_GIBZE	FG02461.1	0.0414	0.9638	0.91	Malate dehydrogenase
tr|I1RPR1|I1RPR1_GIBZE	FG06039.1	0.5779	3.2211	2.65	ATP-citrate lyase
tr|I1RCC4|I1RCC4_GIBZE	FG09896.1	0.2567	2.466	2.21	Isocitrate lyase

Protein peptides were detected using LC–MS/MS as described in the “Materials and methods” section. Accession # is the number in UniProt. Data marked with an asterisk (*) are quoted from Suzuki et al. (2013)

## Discussion

We reported that except for pyruvate, metabolites belonging to the glycolytic system were increased in *F. asiaticum* cells producing DON in previous papers (Suzuki et al. 2013). Because the amounts of mRNAs encoding enzymes belonging to the glycolytic system did not increase, it seemed that the metabolites were increased by something other than gene expression changes, such as protein degradation being suppressed or protein activity being increased.

Among the enzymes belonging to the glycolytic system, the peptide contents of three proteins, ATP-dependent 6-phosphofructokinase, 2,3-bisphosphoglycerate-independent phosphoglycerate mutase and pyruvate kinase, were increased by the addition of agmatine. 2,3-bisphosphoglycerate-independent phosphoglycerate mutase is a metalloenzyme that is found particularly in eubacteria and higher plants (Li et al. 2011). Elucidation of the role of this enzyme in the flow of the glycolysis system and DON production in *Fusarium* spp. will be the subject of a future study.

**Fig. 2:**
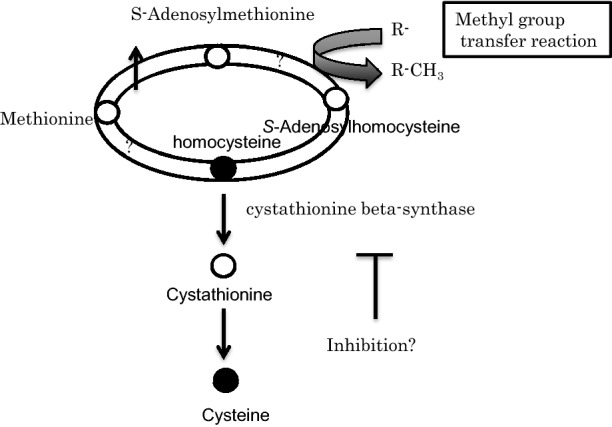
Changes in the methionine cycle after addition of agmatine. ○↑: Up regulated by agmatine. ●↓: Down regulated by agmatine

Phosphofructokinase (PFK) is an enzyme that acts on fructose-6-phosphate and catalyzes one of the key regulatory and rate-limiting steps of glycolysis (Mor et al. 2011). PFK1 converts fructose-6-phosphate to fructose 1,6-phosphate (F-1, 6-BP) and PFK2 converts it to fructose-2, 6-bisphosphate (F-2, 6-BP). There are three types of human PFK1, the muscle, liver and platelet types, which are called PFKM, PFKL and PFKP, respectively (Webb et al. 2015). PFK2 is a strange protein containing a kinase domain and a phosphatase domain, and has been reported to contribute to the Warburg effect of cancer cells in recent years (Ros and Schulze 2013). It is believed that in cancer cells, the TIGAR protein is down regulated by the loss of p53 function, thereby up regulating F-2, 6-BP (Bensaad et al. 2006). As a result, the level of F-2, 6-BP remains high in the tumor and is thought to act as a strong positive stimulus for FPK1. PFK2 is encoded by four different genes (pfkfb1, pfkfb2, pfkfb3 and pfkfb4) in vertebrates. In particular, PFKFB3 exhibits significant kinase activity, enhancing the F-2, 6-BP level and glycolysis efflux (Yalcin et al. 2009). Arginine-methylated PFKFB3 activates glycolytic flux by increasing F-2, 6-BP, an allosteric activator of PFK1 (Mor et al. 2011). As metabolites of the methionine cycle were increased by the addition of agmatine, there is a high possibility that *S*-adenosylmethionine in this metabolic pathway acts as a donor of the methyl group in protein methylation. Our transcriptome and proteome results revealed that the mRNA and protein contents of cystathionine synthase were also down regulated by agmatine addition. These results mean that metabolites necessary for methylation are increased by agmatine. A model of the methionine cycle when agmatine is added is shown in Fig. 2.

In *Aspergillus niger*, citrate resistant PFK1 was activated to a high level by allosteric activators (Mesojednik and Legiša 2005; Mlakar and Legiša 2006; Capuder et al. 2009). These kinetic properties were attributed to the 49 kDa subunit, which is a relatively small PFK1 molecule because other eukaryotic PFK1s are about 85 kDa. Further studies showed that a shorter 49-kDa fragment was formed by a two-step posttranslational modification of the native 85-kDa enzyme (Dunaway 1983; Poorman et al. 1984). In this experiment, F-2, 6-BP could not be detected by metabolomic analysis (data not shown) and the presence of PFK2 could not be detected by proteomic analysis either. However, because the FKP1 peptide remained at a high level during DON production, it is possible that some posttranslational modifications may have occurred.

Pyruvate kinase (EC 2.7.1.40) is the most complex regulatory enzyme of glycolysis, catalyzing the third irreversible step of glucose consumption, and normally controls the outflow from the pathway in eukaryotes. This final step yields ATP and pyruvate. The pyruvate kinase of higher animals has four isozymes, L type, R type, M1 type and M2 type, which are expressed tissue-specifically (Imamura and Tanaka 1982). Among these isoforms, pyruvate kinase M2 (PKM2) is gaining attention. PKM2 functions in glycolysis in a tetramer state and produce pyruvic acid by transferring phosphate from phosphoenolpyruvate to ADP. Dimeric PKM2 is abundant in cancer cells, but PKM2 does not have pyruvate kinase activity in dimer form, causing metabolic changes that may potentially be metabolically advantageous to cancer cells (Gao et al. 2012). In our results, the expression ratio of the pyruvate kinase mRNA was not increased by the addition of agmatine, but the abundance of the detected peptide was increased. Additionally, in metabolomic analysis, pyruvate was decreased and no conversion to lactic acid was detected (data not shown) after agmatine addition.

Both the peptide and mRNA were down regulated for pyruvate dehydrogenase, which produces acetyl-CoA from pyruvate in mitochondria, by agmatine addition. Conversely, the mRNA content of pyruvate dehydrogenase kinase, which phosphorylates pyruvate dehydrogenase and inhibits the influx of pyruvic acid into mitochondria, was up regulated. Unfortunately, the peptide of pyruvate dehydrogenase kinase could not be detected in this experiment. Pyruvate dehydrogenase kinase is an important mitochondrial enzyme that blocks the production of acetyl-CoA by selectively inhibiting the activity of pyruvate dehydrogenase through phosphorylation. Pyruvate dehydrogenase kinase is up regulated in cancer cells, which is considered to be one of the factors contributing to the up regulation of glycolytic metabolism in cancer cells (Zhang et al. 2014).

Pyruvate dehydrogenase kinase 1 deficiency in *F.* *graminearum* resulted in an increase in pyruvate dehydrogenase activity, causing growth retardation and increased pigmentation (Gao et al. 2016). Inhibition of pyruvate dehydrogenase kinase has been suggested as a therapeutic approach for the development of anticancer drugs (Steták et al. 2007). In the future, understanding the activity regulation mechanism of pyruvate dehydrogenase kinase will be necessary for the suppression of DON production.

In *F. asiaticum* cultured with agmatine, both the peptide content and the mRNA expression level of ATP-citrate lyase were high. Because acetyl-CoA cannot pass through the mitochondria, it binds to oxaloacetate to become citric acid and exits the mitochondria into the cytoplasm. ATP-citrate lyase then cleaves citric acid to produce acetyl-CoA for DON synthesis. In addition, peptides of the gamma-aminobutyric acid (GABA) shunt and glyoxylic acid cycle were up regulated by agmatine, which was consistent with transcriptome results described in our previous paper. Among the GABA shunt proteins, the succinate-semialdehyde dehydrogenase protein peptide is considered highly up regulated when GABA formed from agmatine enters the TCA cycle as succinic acid. Oxaloacetate enters the glyoxylate cycle outside the mitochondria and contributes to the production of acetyl-CoA.

As described above, *F. asiaticum* recognizes agmatine as a resistance factor from the plant when acquiring substances necessary for tissue synthesis such as energy, nucleic acids and NADPH through glycolysis and the pentose phosphate pathway. By inhibiting the TCA cycle through the activation of pyruvate dehydrogenase kinase, apoptosis of mitochondria is prevented and proliferation is enabled. This study shows that omics analysis is effective for elucidating the regulatory mechanism of DON production but further detailed research is necessary.

## Abbreviations

DON: deoxynivalenol
iTraq: isobaric tags for relative and absolute quantitation
LC–MS/MS: liquid chromatography coupled with tandem mass spectrometry
PFK: phosphofructokinase
F-1,6-BP: fructose 1,6-phosphate
F-2,6-BP: fructose 2,6-phosphate
PKM2: pyruvate kinase M2
GABA: gamma-aminobutyric acid
